# Supplementary material for: Modeling glioblastoma heterogeneity as a dynamic network of cell states
Source: Mol Syst Biol. 2021 Sep 16;17(9):e10105. doi: 10.15252/msb.202010105 (PMC8444284; doi:10.15252/msb.202010105)
Supplement: Supplementary file 5 — Source Data for Figure 3 [file MSB-17-e10105-s001.zip › Figure3A_sourcedata/GSEA_3065/hallmarks_state2.GseaPreranked.1623416269049/HALLMARK_APICAL_SURFACE.html]

Details for gene set HALLMARK\_APICAL\_SURFACE[GSEA]

|  || Dataset | state2 |
| Phenotype | NoPhenotypeAvailable |
| Upregulated in class | na\_neg |
| GeneSet | HALLMARK\_APICAL\_SURFACE |
| Enrichment Score (ES) | -0.6110104 |
| Normalized Enrichment Score (NES) | -1.760701 |
| Nominal p-value | 0.002739726 |
| FDR q-value | 0.003226529 |
| FWER p-Value | 0.018 |
Table: GSEA Results Summary

  

Fig 1: Enrichment plot: HALLMARK\_APICAL\_SURFACE      
 Profile of the Running ES Score & Positions of GeneSet Members on the Rank Ordered List

  

| PROBE | GENE SYMBOL | GENE\_TITLE | RANK IN GENE LIST | RANK METRIC SCORE | RUNNING ES | CORE ENRICHMENT || 1 | BRCA1 |  |  | 92 | 0.381 | 0.1365 | No |
| 2 | FLOT2 |  |  | 2463 | 0.049 | -0.0964 | No |
| 3 | GAS1 |  |  | 3144 | 0.031 | -0.1568 | No |
| 4 | AKAP7 |  |  | 3313 | 0.027 | -0.1641 | No |
| 5 | ADIPOR2 |  |  | 3549 | 0.023 | -0.1803 | No |
| 6 | HSPB1 |  |  | 3629 | 0.022 | -0.1803 | No |
| 7 | AFAP1L2 |  |  | 4465 | 0.008 | -0.2660 | No |
| 8 | NCOA6 |  |  | 4588 | 0.006 | -0.2767 | No |
| 9 | TMEM8B |  |  | 5293 | -0.005 | -0.3496 | No |
| 10 | GSTM3 |  |  | 5666 | -0.011 | -0.3849 | No |
| 11 | CRYBG1 |  |  | 5950 | -0.016 | -0.4089 | No |
| 12 | SHROOM2 |  |  | 6628 | -0.028 | -0.4701 | No |
| 13 | PLAUR |  |  | 6906 | -0.034 | -0.4863 | No |
| 14 | CX3CL1 |  |  | 7596 | -0.053 | -0.5391 | No |
| 15 | THY1 |  |  | 8139 | -0.074 | -0.5683 | Yes |
| 16 | EPHB4 |  |  | 8413 | -0.090 | -0.5628 | Yes |
| 17 | ADAM10 |  |  | 8868 | -0.132 | -0.5602 | Yes |
| 18 | B4GALT1 |  |  | 8942 | -0.140 | -0.5142 | Yes |
| 19 | SRPX |  |  | 9161 | -0.179 | -0.4685 | Yes |
| 20 | SULF2 |  |  | 9376 | -0.313 | -0.3711 | Yes |
| 21 | APP |  |  | 9412 | -0.426 | -0.2114 | Yes |
| 22 | DCBLD2 |  |  | 9431 | -0.558 | 0.0010 | Yes |
Table: GSEA details [plain text format]

  

Fig 2: HALLMARK\_APICAL\_SURFACE: Random ES distribution      
 Gene set null distribution of ES for **HALLMARK\_APICAL\_SURFACE**
